# Evaluating the effect of the initiative ‘Caring for the Newborn and the Mother at Home’ in Mexico

**DOI:** 10.1017/S1368980020003948

**Published:** 2021-01

**Authors:** Mishel Unar-Munguía, Teresita González de Cosío, Ericka Ileana Escalante-Izeta, Isabel Ferré-Eguiluz, Matthias Sachse-Aguilera, Carolina Herrera

**Affiliations:** 1 Center for Health and Nutrition Research, National Institute of Public Health, Av. Universidad 655 Col. Santa María Ahuacatitlán, Cuernavaca, Morelos 62100, Mexico; 2 Health Department, Universidad Iberoamericana, Mexico City, Mexico; 3 Universidad Iberoamericana, Puebla, Mexico; 4 National Health and Nutrition Officer at UNICEF, Mexico City, Mexico; 5 Operations Officer at World Vision, Mexico City, Mexico

**Keywords:** Home visits, Pregnancy, Newborn, Breast-feeding, Community volunteers, Impact evaluation, Mexico

## Abstract

**Objective:**

The WHO and UNICEF recommend home visits to improve health outcomes for mothers and newborns. We evaluated the effect of home visits by community volunteers during pregnancy and postpartum on breast-feeding practices, women’s knowledge about benefits, beliefs and myths of breast-feeding, obstetric and neonatal warning signs, preparation for childbirth and initial care for newborns, and diarrhoea and respiratory diseases in children.

**Design:**

Community quasi-experimental design. We estimated difference-in-difference models with fixed effects at the community level weighted by propensity score and investigated implementation barriers through focus groups and semi-structured interviews.

**Setting:**

Poor rural communities in Mexico; 48 intervention and 29 control.

**Participants:**

Baseline and follow-up information were reported from two independent cross-sectional samples of women with babies aged between 6 and 18 months (baseline: 292 control, 320 intervention; follow-up: 292 control, 294 intervention).

**Results:**

The intervention increased reports of exclusive breast-feeding in the first 6 months by 24·4 percentage points (pp) (95 % CI: 13·4, 35·4), mothers’ knowledge of obstetric warning signs by 23·4 pp (95 % CI: 9·2, 37·5) and neonatal warning signs by 26·2 pp (95 % CI: 15·2, 37·2) compared to the control group. A non-linear dose–response relation with the number of home visits was found. Diarrhoea and respiratory diseases among children decreased in the intervention *v*. control group but were not statistically significant.

**Conclusions:**

Home visits should be implemented as a complementary strategy to the provision of prenatal and postnatal care in rural communities due to their potential positive effects on the health of mothers and their children.

Worldwide, about 2·4 million newborn babies died during the first month of life in 2019, over a third of neonatal deaths occurred in the first day and three-quarters of deaths occurred in the first week after birth^(1)^. In Mexico, between 1990 and 2018, the neonatal mortality rate declined from 22 to 8 deaths per 1000 live births^(2)^, being higher in entities with a higher level of marginalisation^(3)^. The main causes of neonatal deaths are due to diseases or difficulties in pregnancy, childbirth or the first month after birth^(3)^, while poor breast-feeding practices explain 27 % of infant deaths in the country^(4)^. According to the National Health and Nutrition Survey (ENSANUT), the prevalence of exclusive breast-feeding among children less than 6 months doubled from 14·4 % in 2012 to 28·6 % in 2018 nationally and from 18·5 to 37·4 % in rural dwellings^(5)^. However, these figures are still below the WHO recommendations^(6)^, the Global Nutrition Targets 2025^(7)^ and the recommendations of the National Academy of Medicine and expert groups in Mexico^(8)^.

Although receiving antenatal care during pregnancy reduces the risk of neonatal mortality in low- and middle-income countries^(9)^, 47·9 % of pregnant women in rural areas do not receive adequate prenatal care. This includes receiving a medical consultation during the first trimester of pregnancy, four or more consultations during pregnancy and at least seven of the eight recommended procedures during medical consultations according to Mexican official guidelines^(10)^.

It has been estimated that home visits by community health workers that are complementary to postnatal care in health facilities could prevent between 30 % and 60 % of child deaths in high-mortality settings^(11)^, increase coverage of care, and improve breast-feeding practices and the ability of mothers to identify early warning signs in their newborn, increasing their probability of survival^(11–17)^. The WHO and UNICEF recommend that home visits should be made to mothers during pregnancy and the postpartum period in order to improve health outcomes for mothers and newborns. Community health workers are associated with improved health among the most vulnerable population in Mexico^(18)^, but there is little empirical evidence about their importance as health promotion agents, so documentation and evaluation of their actions is necessary to support scale-up.

Therefore, the objective of this study was to estimate the effect of the intervention ‘Caring for the Newborn and the Mother at Home’^(19)^, which was implemented in poor rural communities in Mexico as a pilot study of home visits by community volunteers (CV) during pregnancy and the first week after postpartum.

We used a cluster quasi-experimental design with an intervention and control group at the community level and studied two independent cross-sectional samples of women with children between 6 and 18 months. We evaluated the reported breast-feeding practices, recognition of warning signs and symptoms for the newborn and the mother during pregnancy and the first week after birth, knowledge about the benefits, beliefs and myths of breast-feeding, preparation for childbirth and initial care newborns should receive, and reporting diarrhoea and respiratory diseases in children. Also, qualitative research was employed to investigate possible implementation barriers of the intervention through focus groups with CV conducting home visits and semi-structured interviews with field work supervisors and doctors.

## Methods

### Intervention description

The intervention ‘Caring for the Newborn and the Mother at Home’ was implemented by the UNICEF and World Vision, a non-profit organisation, from October 2016 to November 2017 in 48 communities in 4 municipalities (Reyes, Zongolica, Texhuacán and Magdalena) located in the Sierra de Zongolica, a mountainous region in the central zone of the State of Veracruz. These communities were selected by World Vision and the Social Security Mexican Institute (IMSS) for being indigenous, having higher than average rates of maternal and neonatal morbidity, having a dispersed population, high and very high levels of marginalisation^(20)^, and being located within 2 h of the municipal head. Similarly, 29 control communities in three municipalities (Tlaquilpa, Rafael Delgado and Soledad Atzompa) were selected for their similar proximity and sociodemographic characteristics to the intervention communities. The number of communities in the intervention and control groups were selected to reach a sample size of 300 women. Fewer control communities were necessary to achieve the same sample size since these communities had, on average, more inhabitants.

All intervention and control communities were part of the ‘PROSPERA’ Social Inclusion Program, which consisted of cash transfers to poor woman conditional to children attending school, health visits for women and their children, and bimonthly health, nutrition and hygiene counselling for women^(21)^. As part of standard care in these communities, women and children received medical attention in the Rural Medical Units (UMR by its Spanish acronym) and Rural Hospitals from IMSS-PROSPERA, where the CV usually identify and refer pregnant and postpartum women and their newborns to the UMR, follow up on their prenatal care, and carry out some home visits to identify risk factors^(22)^.

The WHO/UNICEF original intervention recommends two home visits during pregnancy and three home visits during postpartum; one visit at the day of birth, one visit at the third day and the last visit at the seventh day, and two additional visits for low birth weight babies^(11)^. Since in Mexico many women do not return immediately to their homes after delivery, the intervention was modified and consisted of two home visits to pregnant women and three home visits in the first week after delivery, regardless of their place of birth (hospital or at home). Also, there were two additional home visits for babies with low birth weight and those who had been referred to a UMR due to illness.

**Fig. 1:**
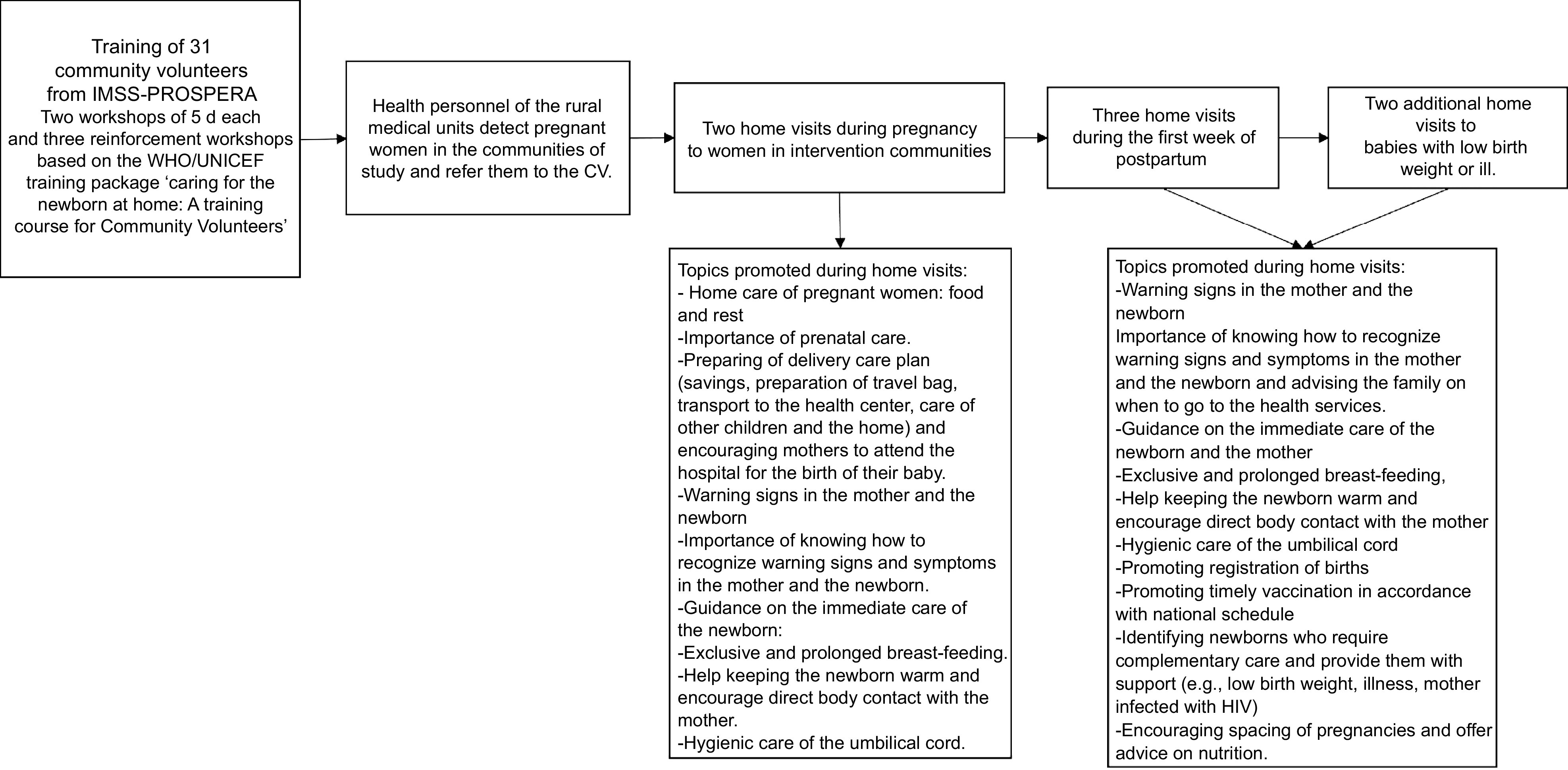
Topics promoted during home visits in pregnancy and postpartum in the study ‘Caring for the Newborn and the Mother at Home’. CV, community volunteers

The original materials were developed by the WHO/UNICEF for personnel training^(19)^ and the intervention has been implemented and validated in other countries^(11,23)^. All materials were translated into Spanish and some messages were adapted to the Mexican context and validated through the practices carried out by the CV during training. Topics promoted during home visits by CV are described in Fig. 1.

The home visits were made by 31 CV, mostly women that were health volunteers belonging to the IMSS-PROSPERA programme. Doctors from the UMR selected CV who were outstanding for their commitment and provided a list of those who would participate in the training. A doctor in the UMR was also trained to follow up on the project implementation, so the CV would consider training as an official topic. The training was provided by the UNICEF, World Vision and IMSS-PROSPERA in two workshops of 5 d each and three reinforcement workshops. After training, CV were invited to implement the intervention in their local UMR and in the areas where the UMR provides coverage. Health personnel of the UMR detected pregnant women in the communities of study and refer them to the CV. World Vision supervised the implementation of the study and the number of home visits realised by each CV, and during the project’s implementation there was an exchange of experiences with field supervisors, to reinforce concepts and clarify doubts.

In addition to being volunteers in this study, the CV participated in the IMSS-PROSPERA programme or supported health centres in their localities in other activities, such as supporting preventive health talks, identifying women and children for the application of vaccines, and inviting and following up women for their monthly appointments, for which they did receive a symbolic payment, usually $26 dollars bimonthly, but was not part of this study’s intervention.

### Impact evaluation design and data collection

The study has a cluster quasi-experimental with a repeated cross-sectional design, since outcomes were not measured on the same women and children before and after the intervention. Two independent cross-sectional samples of women with children between 6 and 18 months of age at baseline and follow-up in the study communities were assessed. Because the outcomes of interest (i.e. the report of breast-feeding practices, identification of warning signs in newborns) could not be measured in the same dyad at baseline and follow-up (at baseline the baby had not been born), the sample of dyads were different in both rounds, although there could be some women surveyed at baseline who had been pregnant and included in the follow-up sample.

Exposure to home visits during pregnancy and postpartum was analysed by intention to treat, which implied that women who lived in the intervention communities were analysed as part of the intervention group and women in control communities as part of the control group.

Eleven interviewers from the Superior Technological Institute of Zongolica, all Nahua speakers, were trained in a 5-d workshop held by the supervisors and the research team. The survey questionnaire was pretested by interviewers during the training before being applied. Women were surveyed face to face and interviewers filled out the survey with the responses. Most of the interviews were conducted in UMR, but the remoteness of some communities to their health centre caused some women not to attend and interviews had to be rescheduled at their home or in public places.

Baseline information was collected from August to December 2016 in control and intervention communities, from all mothers with children between 6 and 18 months of age that agreed to participate. Home visits were implemented from October 2016 to November 2017 with pregnant women living in the intervention communities. After 1 year of implementation and a waiting period of 6 months for children in the follow-up sample to be the same age as the children in the baseline round, information was collected between May and June 2018 in control and intervention communities. At follow-up, a baby that was born at the beginning of the intervention was 18 months old, while a baby born at the end of the intervention period was 6 months old.

To gather additional data, in order to investigate possible barriers to the proper implementation of the intervention, two focus groups were set up, each consisting of six CV in charge of conducting the home visits. Also, three semi-structured interviews were conducted with field work supervisors and the IMSS-PROSPERA doctors.

### Outcome variables

Due to the study design, breast-feeding practices in the first 6 months of life were assessed by recall and not by status quo from the previous day^(24)^. Mothers were asked if they ever breastfed their child, did early breast-feeding, and the duration (days, weeks or months) of any type of breast-feeding, if the child was still being breastfed and if the child received any liquid or solid food (plain water, water with sugar/honey, water with salt and sugar, tea or any brew, oil, formula or non-breast milk, fruit juice, fruits and vegetables, red meat or chicken puree, eggs, any other) before 6 months of age. These liquids and foods are included in the ENSANUT questionnaire to assess breast-feeding practices in Mexico.

The indicator of exclusive breast-feeding was constructed as recommended by WHO^(24)^, considering the number of children that breastfed during the first 6 months and did not receive any other liquid or food before this age. Predominant breast-feeding was constructed similarly, considering if the child received breast milk along with any other non-nutritive liquids such as water or tea.

In addition, information was collected on the women’s knowledge of obstetric and neonatal warning signs in the postpartum period, knowledge about the benefits and myths of breast-feeding and preparation for childbirth, and knowledge about the initial care newborns should receive. Also, we collected information on reports of diarrhoea and respiratory disease in children in the last 2 weeks. A description of indicators is presented in Table 1.

**Table 1:** Outcomes variables evaluated in the study ‘Caring for the Newborn and the Mother at Home’

Breast-feeding practices	
(a) Early initiation of breast-feeding	The percentage of women who reported breast-feeding their child in the first hour of life.
(b) Prevalence of exclusive breast-feeding	The percentage of women who reported to feed their child only with breast milk for the first 6 months of life (allowing the intake of medicines and vitamins).
(c) Prevalence of predominant breast-feeding	The percentage of women who reported to feed their child with breast milk as the predominant source of nourishing and other non-nutritive liquids (water, tea and coffee) for the first 6 months of life.
Knowledge about benefits, beliefs and myths of breast-feeding	
(d) Knowledge about the benefits of breast-feeding	The percentage of women reporting at least one benefit such as ‘the best food for baby’, ‘protects against infections’, ‘protects against diseases when older’, ‘doesn´t cost anything’, ‘it is easy for the baby to digest’ and other (natural food that contains calcium and vitamins helps babies grow fast and healthy and strengthens mother–child ties).
(e) Beliefs and myths of breast-feeding	The percentage of women reporting what should a baby be fed with during the first 6 months of life? And the categories considered are ‘breast milk’, ‘fruit juice’, ‘plain water’, ‘other infusions’, ‘milk’, ‘formula’, and other (‘atole’ beverage with maize and water/milk, broths, jelly, soup, fruits and vegetables).
Knowledge of the warning signs of the mother and the newborn in the postpartum period	
(f) Knowledge of warning signs for the newborn	The percentage of women who mentioned three or more warning signs for the newborn categorised as ‘stop eating’, ‘seizures or attacks’, ‘rapid breathing’, ‘sunken chest’, ‘fever’, ‘low temperature’, ‘yellow soles of the feet’, ‘not moving’, ‘red navel’, ‘navel with pus’, ‘inflamed or irritated skin’, ‘eyes with pus’ and other (doesn’t stop crying, purple skin, vomit, diarrhoea and constipated).
(g) Knowledge of warning signs for the mother	The percentage of women who mentioned three or more warning signs for the mother during the postpartum period categorised as ‘excessive vaginal bleeding’, ‘acute abdominal pain’, ‘attacks’, ‘severe headache’, ‘fever’, ‘rapid breathing or shortness of breath’ and other (high blood pressure, vomiting, ringing in the ears, weakness, dizziness and vaginal discharge).
Knowledge about preparation for childbirth and the initial care for newborns	
(h) Preparation of delivery care plan	The percentage of women that planned to have the baby at the hospital or house, and the percentage of women that prepared before the delivery (savings, preparation of travel bag, transport to the health centre, care of other children and the home).
(i) Knowledge about care of the umbilical cord in the newborn	The percentage of women that reported at least one aspect of care of the umbilical cord considering ‘keep it dry’, ‘clean it’, ‘wash it’ and other (heal it, use waistbands and take care to avoid infections).
(j) Knowledge about hygiene measures in the newborn	The percentage of women that mentioned at least one moment a person should wash her hands when taking care of the baby, including ‘after going to the bathroom’, ‘before entering the baby´s room’, ‘before carrying the baby’, ‘after changing the diaper’ and other (before feeding the baby).
(k) Knowledge about keeping the baby warm	The percentage of women who reported how to keep a baby warm considering the following categories ‘covering with a blanket’, ‘putting on socks and hats’, ‘placing the baby in contact with the mother’ and other (to carry the baby and light a lamp), and the percentage of women who responded from what day a baby born with normal weight or with low birth weight be bathed (first, second, third day or more).
(l) Knowledge about where to go in case of emergency after childbirth	The percentage of women that mentioned to go to ‘the health center’ and ‘the hospital’.
Infectious diseases in children	
(m) Prevalence of diarrhoea in children	The percentage of women that reported their child had diarrhoea in the previous 2 weeks.
(n) Prevalence of respiratory infections in children	The percentage of women that reported their child had respiratory or ear infection in the previous 2 weeks.

### Sample size and statistical power

A total sample size of 300 dyads in each group would have enabled us to estimate, with a statistical power of 80 %, a minimum detectable difference between treatment groups of 8·7 percentage points (pp) in the report of breast-feeding practices, and between 7·4–12·3 pp in the mother’s knowledge of warning signs, beliefs and myths about breast-feeding and initial care of the newborn^(25)^.

### Statistical analysis

We estimated proportions for categorical variables, and means and standard deviations for continuous variables for the main characteristics at the community, household and individual level in baseline and follow-up rounds, for both intervention and control groups.

We assessed the probability (propensity score) for each woman of being in the intervention communities through a probit model^(26)^ adjusted for individual characteristics (child’s sex and age and mother’s age), household characteristics (the number of people in the household; floor, wall, ceiling and roof materials; number of rooms; the presence of a kitchen; availability of drinking water, toilet and electricity), community characteristics (high or very high marginalisation level) and each outcome of interest at baseline.

The analysed outcome variables were (a) breast-feeding practices, (b) knowledge about benefits, beliefs and myths of breast-feeding, (c) knowledge of the warning signs of the mother and the newborn in the postpartum period, (d) knowledge about preparation for childbirth and the initial care for newborns and (e) diarrhoea and respiratory infections in children (Table 1).

We estimated the effect of the intervention on the outcomes through a difference-in-difference model, which compared the difference between follow-up and baseline outcomes in the intervention communities with the difference between follow-up and baseline outcomes in the control communities, using a linear regression with fixed effects at the community level, and weighted by the inverse of the propensity score, and further adjusted for individual, household and community characteristics described above^(26)^.

Additionally, we performed a dose–response sensitivity analysis to assess the association between the reported number of home visits during prenatal and postpartum period (0, <= 1, <= 2, <= 3, <= 4, <= 5, <= 6, <= 7+ visits) and main outcomes.

For all models, we estimated the average marginal effects and reported the intervention effect as percentage point (pp) difference in the outcomes of interest, between intervention and control groups. Differences were statistically significant when *P*-value < 0·05. All statistical analyses were performed in Stata software version 14·2^(27)^.

Regarding the qualitative analysis, the interviews and focus groups were carried out by two previously trained experts. The results were transcribed and their content analysed by an expert in qualitative analysis, using a phenomenology description of the CV’s discourse^(28)^. The analysis was made from the transcripts through speech coding and the categories of analysis focused on the intervention experience, the quality of the intervention, barriers and facilitators for the development of the strategy’s activities, needs of CV and suggestions for programme improvement. No specific programme was used for the analysis.

## Results

### Characteristics of the sample

We collected information from 1198 women with children 6–18 months of age (baseline: 292 control, 320 intervention; follow-up: 292 control, 294 intervention) and analysed information of 1171 dyads with complete information on variables of interest (see online supplementary material, Supplemental Fig. A1).

In the control group, a higher proportion of communities had a very high marginalisation index in comparison to the intervention group, and there were differences at the individual and household level between groups, both at baseline and follow-up (Table 2). After weighting by the propensity score, individual, household and community characteristics were balanced between groups and no statistical differences remained (Table 2 and see online supplementary material, supplemental Fig. A2).

**Table 2:** Sociodemographic characteristics of women and children participating in the study ‘Caring for the Newborn and the Mother at Home’. Before and after propensity score weighting†

Variables	Before propensity score weighting								After propensity score weighting			
	Baseline				Follow-up							
	Intervention		Control		Intervention		Control		Intervention		Control	
	Mean	sd	Mean	sd	Mean	sd	Mean	sd	Mean	sd	Mean	sd
Women and children characteristics	*n* 314		*n* 276		*n* 293		*n* 288		*n* 607		*n* 594	
Mother’s age (years)	26·5	6·0	26·8	6·6	26·7	6·9	26·4	9·4	26·5	7·1	26·5	8·6
Chidren’s age (months)	12·2	3·6	11·5***	3·6	11·7	3·6	11·6**	3·5	11·7	3·9	11·7	4·0
Chidren’s sex (girl = 1)	50·3		57·7*		56·5		50·2		52·6		53·1	
Household characteristics												
Number of persons living in the household												
2–4 (%)	40·0		35·6		53·1		37·3***		41·6		42·3	
5–6 (%)	39·3		38·7		36·4		38·0		36·7		36·9	
7+ (%)	20·6		25·7		10·5		24·7***		21·4		20·9	
Dirt floor (%)	20·6		37·0***		30·2		24·3		27·0		27·1	
Walls (cement, brick, stone and wood) (%)	95·9		98·3*		90·8		89·0		93·7		93·7	
Ceiling (cardboard, rubber, cloth, tires, palma, tejamanil or wood) (%)	5·6		8·2		5·8		7·9		6·0		7·6	
Ceiling (metal, fibreglass and plastic) (%)	73·7		72·9		69·0		65·7		73·2		68·1*	
Ceiling (asbestos sheet) (%)	5·3		3·1		5·1		10·3**		5·0		7·5	
Ceiling (teja) (%)	1·0		5·8**		4·4		3·1		2·6		4·0	
Ceiling (concrete slab, brick or partition) (%)	14·3		9·9*		15·6		13·0		13·1		12·8	
Number of rooms in the house	2·9	1·1	2·8	1·0	2·8	1·0	2·4***	1·1	2·8	1·1	2·8	1·3
Kitchen in the household (%)	93·7		96·2		93·2		90·4		93·1		93·7	
Potable water inside the house (%)	20·0		13·7**		15·0		12·7		15·7		15·9	
Toilette in the household (%)	30·0		25·0		30·6		33·9		31·1		31·1	
Latrine (%)	62·5		67·5		58·5		61·6		61·5		61·5	
Pit, cesspool, blind pit and other (%)	7·5		7·5		10·9		4·5***		7·4		7·4	
Electricity in the house (%)	98·7		98·9		97·9		99·3		98·8		98·8	
Community characteristics												
High marginalisation index (%)	82·2		74·3**		85·3		76·4***		80·2		80·4	
Very high marginalisation index (%)	17·8		25·7**		14·7		23·6***		19·8		19·6	

Differences are statistically significant at ***P < 0·01, **P < 0·05, *P < 0·10.

† Average differences between women and their children in intervention and control communities were estimated through an ordinary least square linear regression clustered at the community level and weighted for the propensity score.

### Implementation of the intervention

The percentage of women in the intervention group that reported at least one home visit by CV during pregnancy increased from 31·9 % in baseline to 73·9 % in follow-up, while in the control group, home visits (standard care) increased from 23·7 to 29·3 %. Women in the intervention group that reported at least one visit during postpartum increased from 18·2 to 66·3 %, very similar to the 67 % reported of supervisors, while in the control group home visits (standard care) increased from 14·8 to 18·4 % in the same period. No home visits were referred by 22 % of women in the intervention group, which could be explained due to some barriers in the implementation, described later in this section.

### Breast-feeding practices

**Fig. 2:**
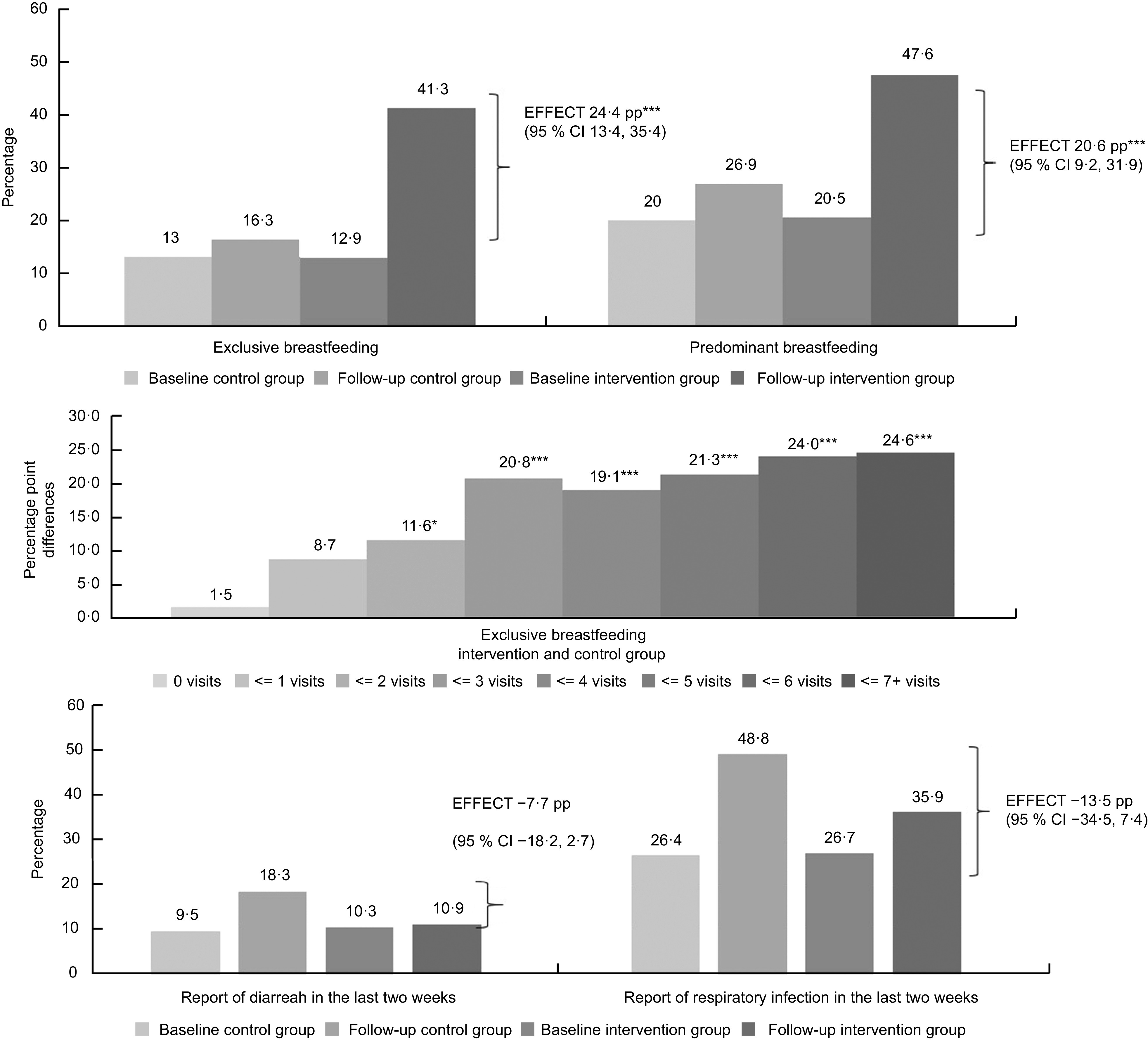
Report of breast-feeding practices and diarrhoea and respiratory diseases among children in intervention and control groups in the study ‘Caring for the Newborn and the Mother at Home’. Difference-in-difference model with fixed effects at the community level weighted by the inverse of the propensity score. Estimates adjusted by sociodemographic variables for the child and the mother. pp: Percentage points estimated through average marginal effects. Respiratory infections include flu, cold, angina, cough, bronchitis or ear infection. Differences are statistically significant at ***P < 0·01, **P < 0·05, *P < 0·10

*The prevalence of exclusive breast-feeding* reported for the first 6 months of life increased by 24·4 pp (95 % CI: 13·4, 35·4) and the prevalence of predominant breast-feeding reported increased by 20·6 pp (95 % CI 9·2, 31·9) in the intervention *v*. the control group (p < 0·001) (Fig. 2). No effect was found on early initiation of breast-feeding in the first hour of birth, which was between 66 % and 68 % in the intervention and control groups (result not shown).

### Infectious diseases in children

The prevalence of diarrhoea and respiratory diseases among children in the previous 2 weeks appeared to be lower in the intervention compared with control group, but no statistical differences were found between the groups (Fig. 2).

### Knowledge about the benefits, beliefs and myths of breast-feeding

The knowledge about the benefits of breast-feeding increased 6·7 pp (95 % CI: 0·8, 12·6) in the intervention *v*. control group (*P* < 0·05). The percentage of women that mentioned at least one benefit of breast-feeding decreased in the control group (Table 3). The beliefs and myths of breast-feeding measured as the percentage of women saying that a baby should be fed plain water, tea, milk or infant formula fell in both groups, but the fall was greater in the control group, so the impact estimates increased for the intervention group (see online supplementary material, Supplemental Table A1). In addition, the percentage of women who thought that babies under 6 months should be fed with ‘atole, broths, jelly, soup, fruits and vegetables’ fell in the intervention group compared to the control group (*P* < 0·001) (see online supplementary material, Supplemental Table A1).

**Table 3:** Knowledge about breast-feeding benefits, warning signs of the mother and the newborn, preparation for childbirth and initial care for newborns among women in the control and intervention communities in the study ‘Caring for the Newborn and the Mother at Home’

	Baseline		Follow-up		Impact† (percentage points) (*n* 1171)	
Indicators	Intervention (*n* 314)	Control (*n* 276)	Intervention (*n* 293)	Control (*n* 288)	%	95 % CI
Breast-feeding benefits						
Mention at least one benefit of breast-feeding (%)	95·8	95·4	95·7	89·9	6·7**	0·8, 12·6
Warning signs for the newborn and the mother reported						
Know three or more warning signs for the newborn (%)	33·5	33·4	39·7	14·8	26·2***	15·2, 37·2
Know three or more warning signs for the mother (%)	31·0	31·9	41·0	17·9	23·4***	9·2, 37·5
What preparations did you make before the delivery? (birth in hospital, clinic or health centre)						
Prepared the things you’d need (%)	79·7	80·1	82·6	71·0	10·5*	−0·4, 21·5
Hat, socks and clothes for the baby (%)	87·9	87·6	90·4	74·6	14·7***	7·2, 22·2
Went to a relative’s or friend’s house near the hospital (%)	5·9	5·6	13·1	0·0	13·0***	5·4, 13·5
Knowledge about hygiene measures in the newborn. When we are taking care of a baby, at what moments should we wash our hands?						
After going to the bathroom (%)	38·0	35·0	43·5	24·9	20·9*	−3·3, 45·0
After changing the diaper (%)	67·0	66·3	60·6	40·5	21·7**	5·1, 38·4
From what day can a baby born with normal weight be bathed?						
First day (%)	63·4	59·6	49·6	45·9	2·4	−8·9, 13·7
Second day (%)	18·8	18·2	31·4	23·0	5·9	−4·4, 16·2
Third day or more (%)	14·5	18·7	13·2	21·5	−5·9	−15·7, 3·8
Knowledge about where to go in case of emergency after childbirth						
Health centre (%)	60·2	63·9	64·7	66·1	3·5	−11·2, 18·2
Hospital (%)	38·8	35·5	34·1	32·7	−3·7	−18·3, 10·8

† Difference-in-differences model with fixed effects at the community level weighted by the inverse of the propensity score.

Estimates adjusted by sociodemographic variables for the child and the mother. Impact was estimated with average marginal effects and presented as percentage point differences between intervention and control communities, statistically significant at ***P < 0·01, **P < 0·05, *P < 0·10.

### Knowledge of warning signs of the mother and the newborn in the postpartum period

There was an increase of mothers’ knowledge of warning signs in newborns, on average, 26·2 pp (95 % CI: 15·2, 37·2) compared to the control group (*P* < 0·001) (Table 3). Also, the intervention increased the mothers’ knowledge of warning signs in women during the postpartum by 23·4 pp (95 % CI: 9·2, 37·5) compared to the control group (*P* < 0·001) (Table 3). For both indicators, knowledge increased among women in the intervention groups and decreased in the control group. Knowledge of specific warning signs is presented in Supplemental Table A2.

### Knowledge about preparation for childbirth and the initial care for newborns

The intervention had a positive impact on the percentage of women that made preparations before the birth of the baby such as ‘prepared the things she’d need’, ‘hat, socks and clothes for the baby’ and ‘she went to the house of a relative or friend near the hospital’ (Table 3).

The knowledge about care of the umbilical cord in the newborn in the intervention and control groups was very high (> 95 %) and did not change after the intervention (results not shown). The intervention had a positive effect on women’s knowledge about hygiene measures in the newborn, such as hand washing ‘after changing the diaper’, but this difference was due to a higher reduction in control group (Table 3).

The intervention did not improve the knowledge about keeping the baby warm, such as placing the baby in contact with the mother so she can transmit her warmth, or delaying the baby’s bath (Table 3 and see online supplementary material, Supplemental Table A3). Concerning the knowledge about where to go in case of emergency after childbirth, the percentage of women that reported the ‘health center’ or the ‘hospital’ was not different between groups (Table 3).

### Sensitivity analysis: dose–response effect of the total number of home visits

We found a positive non-linear dose–response association between the number of home visits and the prevalence of exclusive breast-feeding in the first 6 months of life (Fig. 2) and knowledge of warning signs for the newborn and the mother (see online supplementary material, Supplemental Table A4).

There was no effect on exclusive breast-feeding in the first 6 months of life among women that reported zero, one and two home visits, but receiving between three and seven home visits increased the prevalence of exclusive breast-feeding at diminishing returns, compared to the control group (*P* < 0·001) (Fig. 2). Also, receiving five or more visits increased exclusive breast-feeding compared with two or less home visits (*P* < 0·05) (see online supplementary material, Supplemental Table A4).

### Results of the interviews on the implementation process with community volunteers

The CV felt very satisfied with the intervention. The main perceived benefits were in the orientation and personalised support provided to pregnant women, mainly first-time mothers and teenage mothers. They acknowledged the value of the training they received, as they were not aware of many of the recommendations to prevent maternal mortality and, more importantly, neonatal mortality.

The CV mentioned that, in the UMR, the information that the women receive is very limited and they clearly recognised that the suggestions for maternal and neonatal care are explained in more detail during the home visits. Most of the visits lasted more than an hour, reflecting the care taken by the volunteers not only to offer information but to empathise with the beneficiaries and their families. Mention was made of the need to offer more support and attention to women who were single mothers, since they often lacked the approval and support of their relatives.

Another of the contributions identified was that the pregnant women learned to plan activities around the upcoming childbirth. The contribution of the orientation was particularly noted in terms of savings, identifying a vehicle for getting to the hospital in advance and identifying a shelter where mothers would stay before giving birth in the company of a family member.

In the opinion of the volunteers, other contributions of the programme focus on the creation of links with other localities, their residents and families who were given support during the pregnancy. At the beginning of the programme, the CV felt afraid or embarrassed to initiate the visits, so they began by visiting pregnant women who were relatives or close friends. Subsequently, this fear disappeared and they visited the women who were assigned to them with greater confidence. Several CV reported remaining in contact with the participants and visiting the children from time to time, at the request of the mothers themselves.

There were very special cases where the CV accompanied the women to the hospital, even during the moment of delivery. They helped facilitate the relationship in the hospital, with nurses and doctors. Cases were reported of breast-feeding and health complications. The volunteers themselves solved a number of problems by giving advice on breast-feeding, essentially in cases of low milk supply, or problems with the nipples. They taught postures for breast-feeding the child, massages to stimulate the milk supply and rubbing the nipple to help the babies suck properly. Women were also taught to use a breast pump in specific cases (breast pumps were not provided as part of the intervention).

With regard to the material used, they highlighted the great support provided by the manual and the way it was structured through stories. Through the questions and reflection on the cases read, it was possible to make the best recommendations, especially to women who do not know how to read or write. The cards were used to identify the stage of pregnancy in order to tailor the recommendations to the stage.

With regard to the self-evaluation of their work, the CV said that they were not very satisfied since they did not manage to make all the planned visits. No one evaluated their work as 10 out of 10, since they acknowledge not having been fully involved in their work, although they acknowledge having done everything possible.

Barriers identified to fulfilling their job were the following:

- Lack of accurate information about the date of birth of the children, with the purpose of making the postpartum visits at the right time (caused by the lack of communication with nurses and the families of the beneficiary women).
- Rainy weather, although this did not stop them making their visits, unless the roads were blocked or the river overflowing.
- Failing to attend to their own households in order to provide attention to others, which involves a lot of organisation in order not to abandon their own families.
- Perception that work in the most remote localities puts their safety and women’s health at risk (due to the difficulty of access).
- Presence of dangerous animals in certain locations (dogs or jaguars).
- Hunger, given that they did not have time to eat on the days they made home visits, until they arrived at their homes at night (they report having gone hungry).
- Workload: they preferred to make as many visits as possible in a single day so as not to have to leave their homes so many days each week.
- Criticism: they were criticised by other women in their communities for accepting several positions at the same time and for ‘walking around’ (paying little attention to their own homes).
- Several volunteers decided to continue accompanying pregnant women and supplying them with information from the programme, but only those who lived in their own community. This suggests that women living in very remote areas did not receive the same orientation and support from the programme.

Some recommendations that the CV offer for future interventions:

- Provide blood pressure monitors.
- Provide monetary support for transportation, especially for activities that require visits to another community.
- Look for more people who want to volunteer and not fill too many programmes with the same volunteers from one community.

## Discussion

We find that the intervention ‘Caring for the Newborn and the Mother at Home’ had a positive effect that was highly significant in reports of exclusive and predominant breast-feeding in the first 6 months of the baby’s life. It was also effective at increasing knowledge about the benefits of breast-feeding, the knowledge of obstetric and neonatal warning signs, the hygienic care of the newborn and preparation for the birth. Also, we found a dose–response relation at decreasing rates between the number of home visits, breast-feeding and knowledge of warning signs. The results suggest that the intervention has the potential to reduce the prevalence of diarrhoea and respiratory illness in children, although it was not statistically significant – all of which are mechanisms that have the potential to reduce maternal and neonatal mortality.

The cluster quasi-experimental design with intervention at the community level and repeated cross-sectional independent samples of women and their children is a valid impact evaluation design^(29)^ that has been used in other similar studies and evaluations of health and nutrition interventions at the community level^(14,30,31)^. Furthermore, differences between groups were reduced through weighting by a propensity score^(26)^ which increases the validity of the estimations.

Meanwhile, breast-feeding counselling, an activity very similar to that carried out in these home visits, which includes the community support step of the Baby-friendly Hospital Initiative^(32)^, has proven to be an effective intervention to improve breast-feeding practices in the long term^(16,17,33,34)^. It also reduces diarrhoea incidence among infants^(34)^, supporting the results found in this study.

There is evidence that training community health workers for providing a continuum of care to pregnant women and newborn improves survival among these groups^(23)^. In countries with high child mortality rates, home visits increase key practices such as early initiation of breast-feeding, exclusive breast-feeding, skin-to-skin contact, delaying the first bath, and hygienic practices such as washing hands with soap and water and healing the umbilical cord in hygienic conditions^(11,13–17)^. Also, home visits have shown to be effective in reducing neonatal mortality due to sepsis^(12)^, hypothermia and neonatal mortality^(14)^.

Likewise, there is evidence that exclusive breast-feeding for the first 6 months of life reduces the risk of infectious diseases in children, as well as the risk of death in children in the first year of life^(35)^, which explains one of the biological mechanisms through which the intervention could reduce the prevalence of diarrhoea and respiratory illness among children.

However, there are some limitations of the study. Since we analysed two independent cross sections of women, any differences in unmeasured characteristics that could be associated with the analysed outcomes could bias the results. Although we reduced differences between comparison groups using a propensity score technique to balance observed characteristics at the individual, household and community level, any difference in non-measured characteristics such as women’s ethnicity, education level, civil status, number of children, type of delivery and employment status that have been associated with breast-feeding practices in Mexico could bias the results. However, the probability that this occurred is minimal, since these women share cultural and sociodemographic characteristics.

Also, we measured breast-feeding practices by recall and not by status quo as recommended by WHO^(24)^, which could underestimate breast-feeding indicators due to memory bias^(36)^. However, we expect that the recall bias was not differential between groups. Moreover, baseline prevalence of breast-feeding in the studied communities was similar to those estimated by ENSANUT^(37)^, which used status quo indicators.

Some outcomes deteriorated between baseline and follow-up in the control group, principally those related to knowledge of newborn and obstetric warning signs. Deterioration of antenatal care knowledge among pregnant women after an intervention was implemented is also reported in another trial^(38)^. While the possibility that differences between women in control group could remain, the deterioration effect was not seen on all analysed outcomes, so the results could suggest that the intervention preserved knowledge from deterioration over time.

The design of the intervention does not make it possible to fully exclude the findings due to the Hawthorne effect^(39,40)^. It is possible that mothers have reported better practices because they knew – due to the home visits – that they should do so and not because of the positive effect of the intervention. However, it is likely that this effect does not fully explain the positive results on breast-feeding, given that there were other outcomes that did not produce positive reports, such as early initiation of breast-feeding.

Besides, we could not estimate the effect of the intervention on maternal and neonatal mortality, since a very large sample size was required. Instead, we reported positive effects on breast-feeding practices and the knowledge of warning signs in newborns and mothers, which have the potential to reduce neonatal mortality. Providing information to mothers empowers them to make better health decisions, as previously documented with indigenous women in Mexico^(41)^.

Likewise, the implementation had some limitations. The CV carried out the home visits in adverse conditions due to the terrain, the climate and the dispersion of the homes in the communities under study. These factors caused difficulties, since the CV not only visited their own community but neighbouring communities as well, which involved transport costs, though these may be diminished as more volunteers are trained, eliminating the need to travel longer distances.

Another clear limitation, and one to be considered in future planning sessions, is that there are places like the studied communities where mothers go to stay in special shelters immediately before and/or after giving birth, which prevented the full implementation of the intervention immediately after birth. The fact that the CV did not have a system for identifying the expected date of birth of the baby made it difficult to locate the mothers in time.

Although it was a pilot study, one-fifth of women in the intervention group did not receive any home visit due to the implementation barriers already mentioned. However, almost all women received one visit during pregnancy, two thirds received at least one postpartum visit and 59 % received the expected number of visits which makes the findings plausible. This evaluation shows the effectiveness of the intervention in real conditions, so its potential effect may be greater if implementation and coverage are improved. Many home visit programmes around the world face this challenge, which must be addressed to reach the most vulnerable population^(42)^.

We recommended (1) to scale up the intervention and increase coverage in communities with high poverty levels, in which a similar effect would be expected in the indicators under analysis, (2) to estimate the cost and effectiveness of the intervention if a public health institution performs all the training and implementation to determine the viability of their scale, (3) to implement home visits only in the CV’s community of residence to reduce time and transport costs and (4) to analyse the feasibility of granting economic support to the CV, so that they can cover their transportation and food expenses.

Since the PROSPERA programme and its health component no longer exists in the country, the most vulnerable populations are at risk of not receiving primary health care services. Home visits during pregnancy and postpartum could be a way to reach pregnant women and children in highly marginalised rural areas to ensure they receive prenatal care in health facilities. ‘Caring for the Newborn and the Mother at Home’ should be implemented by health institutions that have trained and managed community health workers, such as the IMSS-Bienestar programme, which is part of the Mexican Social Security Institution and provides health services to people living in marginal rural and urban areas^(43)^. As part of the recent transformation of the health system in Mexico, priority will be given to community prevention, so home visits must be an essential intervention that can be granted as part the new Health Institute for Wellbeing (INSABI), that recently replaced Seguro Popular, whose objective is to provide free medical services to the population without social security^(44)^.

## Conclusions

The impact evaluation shows a positive effect of home visits during pregnancy and postpartum on women’s knowledge of obstetric and neonatal warning signs and in reports of exclusive and predominant breast-feeding in the first 6 months of life. These are mechanisms that have the potential to reduce neonatal mortality. Home visits should continue to be implemented as a complementary strategy to the provision of postnatal care in marginalised communities due to their potential positive effects on the health of mothers and their children.

## Supplementary material

For supplementary material accompanying this paper visit https://doi.org/10.1017/S1368980020003948. click here to view supplementary material

## References

[1] UNICEF (2020) The neonatal period is the most vulnerable time for a child. https://data.unicef.org/topic/child-survival/neonatal-mortality/ (accessed September 2020).

[2] UNICEF, World Health Organization & Bank TW (2019) Levels and Trends in Child Mortality: Report 2019. https://data.unicef.org/resources/levels-and-trends-in-child-mortality/ (accessed September 2020).

[3] Hernández-Bringas H & Narro-Robles J (2019) Mortalidad infantil en México: logros y desafíos (Infant mortality in Mexico: achievements and challenges). Papeles de población 25, 17–49.

[4] Colchero MA , Contreras-Loya D & Lopez-Gatell HGdC (2015) The costs of inadequate breastfeeding of infants in Mexico. Am J Clin Nutr 101, 579–586. 25733643 10.3945/ajcn.114.092775

[5] INSP (2020) Results from the National Health and Nutrition Survey 2018. National Institute of Public Health Mexico. https://ensanut.insp.mx/encuestas/ensanut2018/doctos/informes/ensanut_2018_presentacion_resultados.pdf (accessed March 2020).

[6] The World Health Organization (WHO) (2020) The World Health Organization’s infant feeding recommendation. http://www.who.int/nutrition/topics/infantfeeding_recommendation/en/ (accessed March 2020).

[7] The World Health Organization (WHO) (2019) Global Targets 2025. To Improve Maternal, Infant and Young Child Nutrition. World Health Organization. www.who.int/nutrition/topics/nutrition_globaltargets2025/en (accessed September 2020).

[8] González de Cosío T , Hernández-Cordero S , Rivera-Dommarco J et al. (2017) Recommendations for a multisectorial national policy to promote breastfeeding in Mexico: position of the National Academy of Medicine. Salud Publica Mex 59, 106–113. 28423117 10.21149/8102

[9] Doku DT & Neupane S (2017) Survival analysis of the association between antenatal care attendance and neonatal mortality in 57 low-and middle-income countries. Int J Epidemiol 46, 1668–1677. 29040531 10.1093/ije/dyx125 PMC5837573

[10] Heredia-Pi I , Servan-Mori E , Darney BG et al. (2016) Measuring the adequacy of antenatal health care: a national cross-sectional study in Mexico. Bull World Health Organ 94, 452. 27274597 10.2471/BLT.15.168302 PMC4890208

[11] The World Health Organization (WHO) (2009) Home visits for the newborn child: a strategy to improve survival: WHO/UNICEF joint statement. https://apps.who.int/iris/bitstream/handle/10665/70002/WHO_FCH_CAH_09.02_eng.pdf?sequence=1 (accessed September 2020). 24809117

[12] Bang AT , Bang RA , Baitule SB et al. (1999) Effect of home-based neonatal care and management of sepsis on neonatal mortality: field trial in rural India. Lancet 354, 1955–1961. 10622298 10.1016/S0140-6736(99)03046-9

[13] Memon ZA , Khan GN , Soofi SB et al. (2015) Impact of a community-based perinatal and newborn preventive care package on perinatal and neonatal mortality in a remote mountainous district in Northern Pakistan. BMC Pregnancy Childbirth 15, 106. 25925407 10.1186/s12884-015-0538-8 PMC4446857

[14] Baqui AH , Darmstadt GL , Williams EK et al. (2008) Effect of community-based newborn-care intervention package implemented through two service-delivery strategies in Sylhet district, Bangladesh: a cluster-randomised controlled trial. Lancet 371, 1936–1944. 18539225 10.1016/S0140-6736(08)60835-1

[15] Olds DL , Kitzman H , Knudtson MD et al. (2014) Effect of home visiting by nurses on maternal and child mortality. JAMA Pediatr 168, 800. 25003802 10.1001/jamapediatrics.2014.472 PMC4235164

[16] Perez K , Patterson J , Hinshaw J et al. (2018) Essential Care for Every Baby: improving compliance with newborn care practices in rural Nicaragua. BMC Pregnancy Childbirth 18, 371. 30208870 10.1186/s12884-018-2003-y PMC6136183

[17] Chanani S , Waingankar A , Shah More N et al. (2018) Participation of pregnant women in a community-based nutrition program in Mumbai’s informal settlements: effect on exclusive breastfeeding practices. PLoS One 13, e0195619. 29621355 10.1371/journal.pone.0195619 PMC5886586

[18] Balcazar H , Perez-Lizaur AB , Izeta EE et al. (2016) Community health workers-promotores de salud in Mexico. J Ambul Care Manage 39, 12–22. 26650742 10.1097/JAC.0000000000000096

[19] World Health Organization (WHO) (2015) Caring for the newborn at home. Training materials. https://www.who.int/maternal_child_adolescent/documents/caring-for-the-newborn-at-home/en/ (accessed November 2019).

[20] CONAPO (2010) National Council of Population and Housing. Marginalization index at community level. http://www.conapo.gob.mx/es/CONAPO/Indice_de_Marginacion_por_Localidad_2010 (accessed July 2016).

[21] Dávila Lárraga LG (2016) How Does Prospera Work? Best Practices in the implementation of Conditional Cash Transfer Programs in Latin America and the Caribbean. Washington DC: Inter-American Development Bank.

[22] IMSS (2016) IMSS-PROSPERA program. http://www.imss.gob.mx/sites/all/statics/pdf/informes/20152016/12-Cap08.pdf (accesed March 2020).

[23] Aboubaker S , Qazi S , Wolfheim C et al. (2014) Community health workers: a crucial role in newborn health care and survival. J Glob Health 4, 020302. 25520788 10.7189/jogh.04.020302 PMC4267086

[24] World Health Organization (WHO) (2010) Indicators for Assessing Infant and Young Child Feeding Practices: Part 2: Measurement. Geneva: World Health Organization.

[25] Murray DM (1998) Design and Analysis of Group-Randomized Trials, vol. 29. New York: Oxford University Press.

[26] Stuart EA , Barry CL , Huskamp HA et al. (2014) Using propensity scores in difference-in-differences models to estimate the effects of a policy change. Heal Serv Outcomes Res Methodol 14, 166–182. 10.1007/s10742-014-0123-z PMC4267761 25530705

[27] StataCorp (2014) Stata Statistical Software: Release 14.2 [Computer Software]. College Station, TX: StataCorp LP.

[28] Heidegger M (1988) The Basic Problems of Phenomenology. Bloomington: Indiana University Press.

[29] Khandker SR , Koolwal GB & Sammad HA (2010) Handbook on Impact, Quantitative Methods and Practices. Washington, DC: World Bank.

[30] Ramírez-Luzuriaga MJ , Unar-Munguía M , Rodríguez-Ramírez S et al. (2016) A food transfer program without a formal education component modifies complementary feeding practices in poor rural Mexican communities. J Nutr 146, 107–113. 26561408 10.3945/jn.115.215962

[31] Pramanik S , Ghosh A , Nanda RB et al. (2018) Impact evaluation of a community engagement intervention in improving childhood immunization coverage: a cluster randomized controlled trial in Assam, India. BMC Public Health 18, 534. 29688845 10.1186/s12889-018-5458-x PMC5913885

[32] Pérez-Escamilla R , Martinez JL , Segura-Pérez S et al. (2016) Impact of the Baby-friendly Hospital Initiative on breastfeeding and child health outcomes: a systematic review. Matern Child Nutr 12, 402–417. 26924775 10.1111/mcn.12294 PMC6860129

[33] Morrow AL , Shults J , Butterfoss FD et al. (1999) Efficacy of home-based peer counselling to promote exclusive breastfeeding: a randomised controlled trial. Lancet 353, 1226–1231. 10217083 10.1016/S0140-6736(98)08037-4

[34] Chapman DJ , Morel K , Anderson AK et al. (2010) Breastfeeding peer counseling: from efficacy through scale-up. J Hum Lact 26, 314–326. 20715336 10.1177/0890334410369481 PMC3115698

[35] Sankar MJ , Sinha B , Chowdhury R et al. (2015) Optimal breastfeeding practices and infant and child mortality: a systematic review and meta-analysis. Acta Paediatr 104, 3–13. 26249674 10.1111/apa.13147

[36] González-Castell D , González de Cosío T , Rodríguez-Ramírez S et al. (2016) Early consumption of liquids different to breast milk in Mexican infants under 1 year: results of the probabilistic National Health and Nutrition Survey 2012. Nutr Hosp 33, 14–20. 27019236 10.20960/nh.v33i1.9

[37] González de Cosío T , Escobar-Zaragoza L , Gonzalez-Castell LD et al. (2013) Infant feeding practices and deterioration of breastfeeding in Mexico. Salud Publica Mex 55, Suppl. 2, S170–S179. 24626693

[38] Nuraini E & Parker E (2005) Improving knowledge of antenatal care (ANC) among pregnant women: a field trial in central Java, Indonesia. Asia Pacific J Public Health 17, 3–8. 10.1177/101053950501700102 16044824

[39] Adair JG (1984) The Hawthorne effect: a reconsideration of the methodological artifact. J Appl Psychol 69, 334.

[40] Aidam BA , Perez-Escamilla R & Lartey A (2005) Lactation counseling increases exclusive breast-feeding rates in Ghana. J Nutr 135, 1691–1695. 15987851 10.1093/jn/135.7.1691

[41] Amaya-Castellanos CI , Shamah-Levy T , Escalante-Izeta EI et al. (2019) Empoderamiento y búsqueda de atención en salud: un factor ignorado de la mortalidad materna en una comunidad indígena mexicana (Empowerment and seeking health care: an ignored factor of maternal mortality in a Mexican indigenous community). Glob Health Promot 27, 166–174. 31062659 10.1177/1757975918821052

[42] McPherson R & Hodgins S (2018) Postnatal home visitation: lessons from country programs operating at scale. J Glob Health 8, 010422. 29977530 10.7189/jogh.08.010422 PMC6005634

[43] IMSS-Bienestar (2020) IMSS-Bienestar. http://www.imss.gob.mx/imss-bienestar (accessed March 2020).

[44] Ministry of Health Mexico (2020) Atención Primaria de Salud Integral e Integrada APS-I Mx: la propuesta metodológica y operativa (Comprehensive and Integrated Primary Health Care APS-I Mx: the methodological and operational proposal). http://www.sidss.salud.gob.mx/site2/docs/Distritos_de_Salud_VF.pdf (accessed March 2020).

