# Supplementary material for: Evaluating the effect of the initiative ‘Caring for the Newborn and the Mother at Home’ in Mexico
Source: Public Health Nutr. 2020 Oct 7;24(1):157–68. doi: 10.1017/S1368980020003948 (PMC7754566; doi:10.1017/S1368980020003948)
Supplement: Supplementary file 1 [file S1368980020003948sup001.docx]

Supplementary figure A1. Flow diagram of the sample size of women with children between 6 and 18 months of age living in control and intervention communities in the study “Caring for the newborn and the mother at home”.

Women in

intervention group

**Follow-up**

48 communities

n=302

398

Women participating in the interviews (n=1,317)

N=714

Women in

control group

**Baseline**

28 communities

n=316

Women in

intervention group

**Baseline**

66 communities

n=398

398

**Intervention group Baseline**

64 communities

Women with children 6-18 months of age **n=320**

**Exclusion (n=8)**

Children <6 months of age

n=1

Children > 18 months of age

n=7

**Control group Baseline**

28 communities

Women with children 6-18 months of age **n=292**

**Exclusion (n=24)**

Children <6 months of age

n=8

Children > 18 months of age

n=16

Women in

control group

**Follow-up**

29 communities

n=301

**Control group Follow-up**

29 communities

Women with children 6-18 months of age **n=292**

**Intervention group Follow-up**

47 communities

Women with children 6-18 months of age **n=294**

**Exclusion (n=9)**

Children <6 months of age

n=3

Children > 18 months of age

n=6

**Exclusion (n=78)**

Children <6 months of age

n=37

Children > 18 months of age

n=41

**Women with children**

**6-18 months of age**

**(n=1,198)**

N=714

Incomplete sociodemographic information

(n=27)

N=714

**Analyzed sample**

**Women with children**

**6-18 months of age**

**(n=1,171)**

N=714

Supplementary figure A2. Propensity score distribution between treatment groups before and after propensity score matching


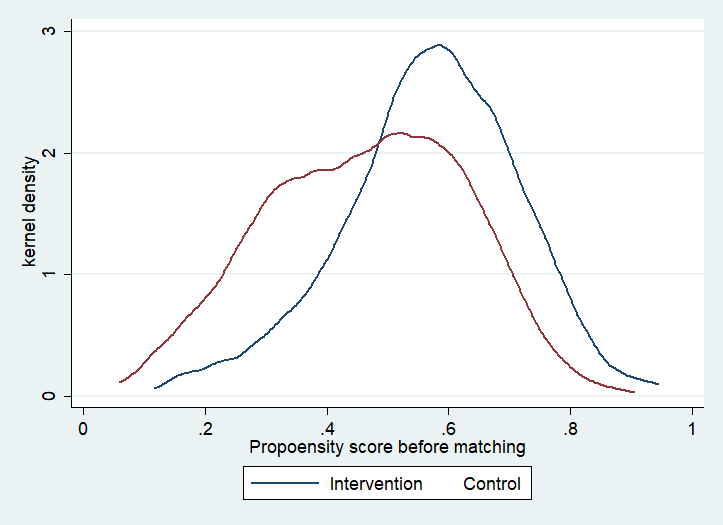


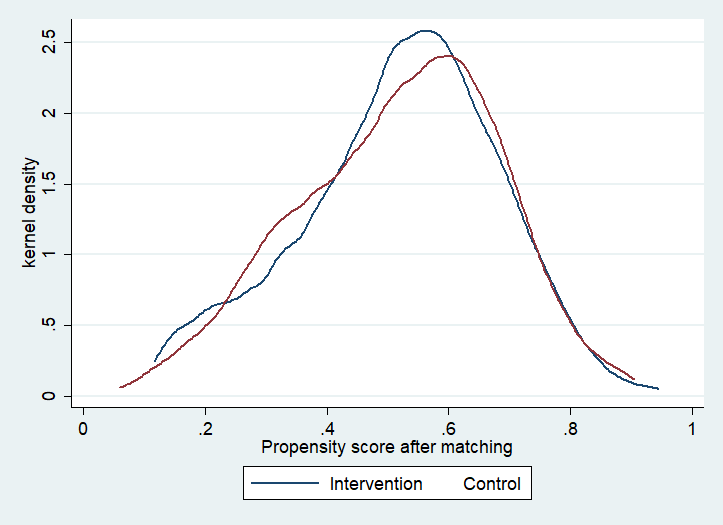


Supplementary table A1. Knowledge about the benefits, beliefs and myths of breastfeeding among women in the control and intervention communities of the study “Caring for the newborn and the mother at home”

|  | Baseline | | Follow Up | | Impact† |
| --- | --- | --- | --- | --- | --- |
| *Indicators* | Intervention  (n= 314) | Control  (n= 276) | Intervention  (n= 293) | Control  (n= 288) | (percentage points) (n=1,171) |
| Mention at least one benefit of breastfeeding (%) | 95.8 | 95.4 | 95.7 | 89.9 | 6.7**  (0.8, 12.6) |
| *Breastfeeding benefits* | | | | | |
| It is the best food for the baby (%) | 72.6 | 78.2 | 70.9 | 58.0 | 20.9***  (10.0, 31.8) |
| Protects against infections (%) | 68.2 | 54.0 | 58.5 | 51.1 | -5.4  (-19.4, 8.6) |
| Protects against disease when older (%) | 51.8 | 45.8 | 58.3 | 28.9 | 23.1***  (9.7, 36.5) |
| Doesn't cost anything (%) | 1.25 | 21.5 | 6.0 | 5.3 | 19**  (4.4, 34.5) |
| It is easy for the baby to digest (%) | 2.7 | 11.8 | 11.3 | 4.2 | 14.5***  (4.9, 24.1) |
| Other ^b^ (%) | 10.0 | 6.5 | 15.1 | 24.6 | -14.1***  (-22.6, -5.6) |
| *What should a baby be fed with during the first 6 months of life?* | | | | | |
| Breast milk (%) | 86.9 | 97.7 | 95.7 | 88.5 | 17.9***  (10.4, 25.3) |
| Fruit juice (%) | 13.4 | 32.8 | 10.7 | 14.2 | 17.4  (-6.0, 40.8) |
| Plain water | 3.1 | 13.6 | 3.4 | 3.9 | 11.6**  (1.4, 21.8) |
| Water, tea or other infusion with salt and/or sugar (%) | 7.9 | 33.1 | 2.3 | 6.4 | 22.0***  (7.1, 36.9) |
| Milk (not maternal or formula) (%) | 4.2 | 3.6 | 0.7 | 3.6 | -3.5  (-8.0, 1.0) |
| Formula (%) | 3.5 | 12.0 | 1.1 | 1.9 | 7.2*  (-0.9, 15.5) |
| Other (atole, broths, jelly, soup, fruits and vegetables) (%) | 28.2 | 18.4 | 10.3 | 19.9 | -19.8***  (-31.8, 7.7) |

†. Difference in differences model with fixed effects at the community level, weighted by the inverse of the propensity score. Estimates adjusted by sociodemographic variables for the child and the mother. b. natural food, contains calcium, vitamins, helps babies grow fast, healthy, strengthens mother-child ties. c. Differences between intervention and control communities ***p<0.01, **p<0.05, *p<0.10.

Supplementary table A2. Knowledge of warning signs of the mother and the newborn in the postpartum period among women in the control and intervention communities of the study “Caring for the newborn and the mother at home”

|  | Baseline | | | Follow Up | | | Impact† |
| --- | --- | --- | --- | --- | --- | --- | --- |
| *Indicators* | Intervention  (n= 314) | Control  (n= 276) | Intervention  (n= 293) | | Control  (n= 288) | (percentage points)  (n=1,171) | |
| *Know three or more warning signs for the newborn (%)* | 33.5 | 33.4 | 39.7 | | 14.8 | 26.2***  (15.2, 37.2) | |
| *Know three or more warning signs for the mother (%)* | 31.0 | 31.9 | 41.0 | | 17.9 | 23.4***  (9.2, 37.5) | |
| *Warning signs for the newborn reported* | | | | | | | |
| *Stops eating (%)* | 34.4 | 47.4 | 46.2 | | 41.2 | 18.3***  (5.4, 31.2) | |
| *Seizures or attacks (%)* | 2.4 | 4.0 | 3.9 | | 1.7 | 4.0**  (0.7, 7.3) | |
| *Rapid breathing (%)* | 30.4 | 28.7 | 35.2 | | 16.8 | 15.9***  (4.6, 27.3) | |
| *Sunken chest (%)* | 2.5 | 2.2 | 4.5 | | 1.0 | 2.8  (-1.8, 7.4) | |
| *Fever (%)* | 36.1 | 49.4 | 34.8 | | 24.5 | 26.4***  (9.1, 43.7) | |
| *Low temperature (%)* | 6.3 | 7.3 | 11.9 | | 5.3 | 10.0**  (2.2, 17.8) | |
| *Yellow soles of the feet (%)* | 17.5 | 15.3 | 34.4 | | 6.9 | 26.6***  (16.2, 36.9) | |
| *Not moving (%)* | 10.8 | 13.5 | 11.7 | | 10.8 | 3.7  (-6.2, 13.7) | |
| *Red navel (%)* | 5.1 | 5.5 | 2.3 | | 0.5 | 2.8  (-1.9, 7.5) | |
| *Navel with pus (%)* | 4.0 | 1.9 | 2.8 | | 0.9 | 0.8  (-2.5, 4.2) | |
| *Inflamed or irritated skin (%)* | 2.1 | 1.3 | 1.3 | | 1.0 | -0.12  (-3.2, 2.9) | |
| *Eyes with pus (%)* | 0.1 | 1.0 | 2.0 | | 0.1 | 2.9**  (0.0, 5.8) | |
| *Other ^b^ (%)* | 59.7 | 46.9 | 42.8 | | 45.7 | -15.1*  (-32.6, 2.2) | |
| *Warning signs for the women reported* | | | | | | | |
| *Excessive vaginal bleeding (%)* | 67.8 | 52.2 | 70.5 | | 55.4 | 1.8  (-10.0, 13.6) | |
| *Acute abdominal pain (%)* | 21.7 | 32.4 | 26.6 | | 21.9 | 14.2*  (-1.6, 30.1) | |
| *Attacks (%)* | 1.9 | 5.9 | 6.3 | | 3.3 | 8.0*  (-0.6, 16.7) | |
| *Severe headache (%)* | 30.2 | 42.9 | 43.6 | | 26.3 | 29.2***  (10.1, 48.3) | |
| *Fever (%)* | 18.6 | 26.7 | 26.6 | | 9.1 | 26.7***  (16.3, 37.0) | |
| *Rapid breathing or shortness of breath (%)* | 0.9 | 3.0 | 6.6 | | 5.2 | 2.4  (-2.8, 7.7) | |
| *Other ^c^ (%)* | 43.3 | 32.2 | 40.0 | | 27.8 | -0.03  (-15.8, 15.8) | |

†. Difference in differences model with fixed effects at the community level, weighted by the inverse of the propensity score. Estimates adjusted by sociodemographic variables for the child and the mother. b. Doesn’t stop crying, purple skin, vomit, diarrhea, constipated. c. high blood pressure, vomiting, ringing in the ears, weakness, dizziness, vaginal discharge. Significant difference between intervention and control group*** p<0.01, ** p<0.05, * p<0.10.

Supplementary table A3. Knowledge about preparation for childbirth and the initial care for newborns among women in the control and intervention communities of the study “Caring for the newborn and the mother at home”

|  | Baseline | | Follow Up | | | Impact† | |
| --- | --- | --- | --- | --- | --- | --- | --- |
| *Indicators* | Intervention  (n= 314) | Control  (n= 276) | Intervention  (n= 293) | Control  (n= 288) | (percentage points) (n=1,171) | | |
| *Knowledge about hygiene measures in the newborn. When we are taking care of a baby, at what moments should we wash our hands?* | | | | | | | |
| *Mention at least one moment (%)* | 99.1 | 99.8 | 99.6 | 98.2 | 0.9  (-0.9, 2.9) | | |
| After going to the bathroom (%) | 38.0 | 35.0 | 43.5 | 24.9 | 20.9*  (-3.3, 45.0) | | |
| *Before entering the baby's room (%)* | 25.3 | 17.2 | 29.0 | 18.5 | 0.1  (-10.4, 10.7) | | |
| *Before carrying the baby (%)* | 53.2 | 45.5 | 63.0 | 44.9 | 10.5  (-5.6, 26.1) | | |
| After changing the diaper (%) | 67.0 | 66.3 | 60.6 | 40.5 | 21.7**  (5.1, 38.4) | | |
| *Other (Before feeding) (%)* | 41.9 | 48.2 | 30.5 | 48.6 | -11.5  (-29.1, 6.1) | | |
| *Knowledge about keeping a baby warm. How can you keep a baby warm?* | | | | | | | |
| Covering it with a blanket (%) | 88.4 | 89.9 | 78.4 | 79.7 | | | -0.7  (-9.5, 8.0) |
| Putting on socks and hat (%) | 65.2 | 64.4 | 40.4 | 29.2 | | | 10.6  (-6.0, 27.1) |
| Placing it in contact with the mother so she can transmit her warmth (%) | 50.0 | 50.0 | 63.0 | 47.0 | | | 13.5  (-2.6, 29.7) |
| *Other (Carry it, light a lamp, don’t take it outside) (%)* | 6.0 | 5.9 | 6.2 | 9.0 | -2.4  (-8.7, 3.9) | | |
| *From what day can a baby born with normal weight be bathed?* | | | | | | | |
| *First day (%)* | 63.4 | 59.6 | 49.6 | 45.9 | 2.4  (-8.9, 13.7) | | |
| *Second day (%)* | 18.8 | 18.2 | 31.4 | 23.0 | 5.9  (-4.4, 16.2) | | |
| *Third day or more (%)* | 14.5 | 18.7 | 13.2 | 21.5 | -5.9  (-15.7, 3.8) | | |
| *Fourth day or more (%)* | <0.1 | 6.8 | 4.7 | 9.1 | 2.8  (-1.4, 7.2) | | |
| *From what day can a baby born with low birthweight be bathed?* | | | | | | | |
| *First day (%)* | 25.2 | 22.0 | 21.0 | 19.1 | -2.0  (-15.7, 11.7) | | |
| *Second day (%)* | 24.7 | 23.3 | 25.0 | 15.9 | 9.3  (-4.8, 23.4) | | |
| *Third day (%)* | 29.2 | 29.2 | 29.0 | 24.6 | 3.5  (-10.3, 17.2) | | |
| *Fourth day or more (%)* | 21.5 | 28.0 | 25.1 | 39.7 | -9.1  (-24.1, 5.9) | | |
| *Knowledge about where to go in case of emergency after childbirth* | | | | | | | |
| *Health center (%)* | 60.2 | 63.9 | 64.7 | 66.1 | 3.5  (-11.2, 18.2) | | |
| *Hospital (%)* | 38.8 | 35.5 | 34.1 | 32.7 | -3.7  (-18.3, 10.8) | | |
| *Where had you planned to have your baby?* | | | | | | | |
| Hospital (%) | 91.3 | 89.4 | 95.0 | 92.9 | 0.2  (-7.6, 8.0) | | |
| *What preparations did you make before the delivery? (Birth in hospital, clinic or health center)* | | | | | | | |
| Identified a means of transport (%) | 53.9 | 54.8 | 75.3 | 66.6 | 4.7  (-8.3, 17.5) | | |
| Saved money for transport (%) | 71.1 | 61.3 | 81.2 | 70.1 | -3.6  (-16.4, 9.2) | | |
| Saved money for other expenses (%) | 71.1 | 70.7 | 83.5 | 67.2 | 13.4  (-4.4, 31.2) | | |
| Asked someone to take care of your home or other children (%) | 51.1 | 38.1 | 53.5 | 34.5 | 3.5  (-10.5, 17.5) | | |
| Prepared the things you’d need (%) | 79.7 | 80.1 | 82.6 | 71.0 | 10.5*  (-0.4, 21.5) | | |
| Hat, socks and clothes for the baby (%) | 87.9 | 87.6 | 90.4 | 74.6 | 14.7***  (7.2, 22.2) | | |
| Went to a shelter near the hospital (%) | 21.4 | 6.5 | 14.5 | 2.0 | -2.0  (-10.0, 6.1) | | |
| Went to a relative's or friend’s house near the hospital (%) | 5.9 | 5.6 | 13.1 | 0.0 | 13.0***  (5.4, 13.5) | | |
| Other (Other baby items, documents) (%) | 3.7 | 0.7 | 3.2 | 4.2 | -3.6**  (-7.1, 0.0) | | |

†. Difference in differences model with fixed effects at the community level, weighted by the inverse of the propensity score. Estimates adjusted by sociodemographic variables for the child and the mother. Significant difference between intervention and control group*** p<0.01, ** p<0.05, * p<0.10.

Supplementary table A4. Dose-response effect of the reported number of home visits (prenatal and postpartum) with exclusive breastfeeding and knowledge of warning signs among women in the study “Caring for the newborn and the mother at home”. Sensitivity analysis.

|  | Baseline | | Follow Up | | Impact† |
| --- | --- | --- | --- | --- | --- |
| *Number of Visits during prenatal and postpartum* | Intervention | Control | Intervention | Control | (percentage points) |
| *Exclusive Breastfeeding* | | | | | |
| Visits = 0 | 14.3 | 14.1 | 22.7 | 20.0 | 1.5  (-12.4, 15.5) |
| Visits <= 1 | 13.6 | 13.3 | 28.0 | 18.1 | 8.7  (-4.7, 22.1) |
| Visits <= 2 | 13.1 | 13.0 | 30.4 | 18.0 | 11.6*  (-1.6, 24.8) |
| Visits <= 3 | 12.7 | 12.4 | 37.9 | 17.0 | 20.8***  (8.2, 33.3) |
| Visits <= 4 | 13.3 | 12.4 | 37.3 | 16.5 | 19.1***  (6.4, 31.8) |
| Visits <= 5 | 13.0 | 12.3 | 38.6 | 16.1 | 21.3***  (10.0, 32.5) |
| Visits <= 6 | 13.0 | 12.6 | 40.3 | 15.7 | 24.0***  (12.7, 35.3) |
| Visits <= 7+ | 13.0 | 12.9 | 41.0 | 15.8 | 24.5***  (13.7, 35.6) |
| 5 or more visits vs 4 or less visits | 18.1 | 7.7 | 36.1 | 20.8 | 9.7  (-3.2, 22.6) |
| 5 or more visits vs 3 or less visits | 17.4 | 7.8 | 35.7 | 21.4 | 10.5*  (-2.0, 23.1) |
| 5 or more visits vs 2 or less visits | 16.2 | 9.9 | 34.3 | 20.6 | 15.1**  (1.9, 28.4) |
| 5 or more visits vs 1 or less visits | 16.2 | 10.6 | 34.3 | 20.4 | 16.8**  (3.3, 30.4) |
| *Knowledge of warning signs for the newborn.* Know three or more warning signs for the newborn (%) | | | | | |
| Visits = 0 | 33.4 | 31.1 | 36.1 | 17.8 | 16.4  (-10.0, 24.5) |
| Visits <= 1 | 35.4 | 32.7 | 34.5 | 16.4 | 16.0*  (-2.6, 34.6) |
| Visits <= 2 | 34.5 | 34.0 | 34.6 | 15.4 | 19.3**  (1.8, 36.8) |
| Visits <= 3 | 34.6 | 33.6 | 38.1 | 15.3 | 23.8***  (8.9, 38.5) |
| Visits <= 4 | 34.0 | 33.8 | 37.3 | 15.3 | 22.9***  (9.3, 36.4) |
| Visits <= 5 | 33.6 | 33.8 | 38.6 | 14.7 | 25.8***  (13.5, 38.0) |
| Visits <= 6 | 33.9 | 33.6 | 38.9 | 14.8 | 25.4***  (13.9, 36.9) |
| Visits <= 7+ | 33.5 | 33.3 | 39.7 | 14.8 | 26.2***  (15.1, 37.2) |
| 5 or more visits vs 4 or less visits | 37.9 | 29.0 | 33.4 | 21.2 | 16.3**  (0.3, 32.3) |
| 5 or more visits vs 3 or less visits | 37.9 | 29.5 | 33.2 | 21.2 | 17.9**  (1.5, 34.3) |
| 5 or more visits vs 2 or less visits | 36.9 | 30.7 | 33.0 | 20.3 | 21.5**  (4.5, 38.4) |
| 5 or more visits vs 1 or less visits | 37.1 | 30.2 | 34.0 | 20.5 | 19.8**  (2.1, 37.6) |
| *Knowledge of warning signs for the mother.* Know three or more warning signs for the mother (%) | | | | | |
| Visits = 0 | 30.2 | 32.7 | 28.5 | 17.7 | 13.5  (-3.4, 30.5) |
| Visits <= 1 | 31.4 | 32.0 | 31.4 | 17.6 | 14.4*  (-0.8, 29.6) |
| Visits <= 2 | 31.3 | 31.6 | 35.1 | 17.9 | 17.3**  (1.0, 33.6) |
| Visits <= 3 | 31.7 | 31.5 | 38.2 | 17.6 | 20.7**  (5.2, 36.3) |
| Visits <= 4 | 31.4 | 32.2 | 39.0 | 18.2 | 21.1***  (5.4, 36.7) |
| Visits <= 5 | 31.3 | 32.8 | 42.6 | 18.4 | 26.3***  (11.5, 41.0) |
| Visits <= 6 | 31.3 | 32.3 | 41.8 | 17.9 | 25.3***  (11.0, 39.6) |
| Visits <= 7+ | 31.0 | 31.8 | 41.0 | 17.9 | 23.4***  (9.2, 37.5) |
| 5 or more visits vs 4 or less visits | 35.7 | 27.3 | 35.9 | 23.0 | 13.7  (-5.1, 32.5) |
| 5 or more visits vs 3 or less visits | 35.6 | 27.1 | 35.5 | 22.0 | 14.8  (-4.4, 34.0) |
| 5 or more visits vs 2 or less visits | 35.0 | 27.6 | 35.5 | 20.9 | 17.2*  (-1.4, 35.8) |
| 5 or more visits vs 1 or less visits | 34.6 | 28.6 | 34.8 | 20.3 | 19.3**  (0.6, 38.0) |

†. Difference in differences model with fixed effects at the community level, weighted by the inverse of the propensity score. Estimates adjusted by sociodemographic variables for the child and the mother. Impact was estimated with average marginal effects and presented as percentage point differences between intervention and control communities, statistically significant at ***p<0.01, **p<0.05, *p<0.10.
